# Anterior Horn Cell Disease in Adulthood: Unmasking Spinal Muscular Atrophy Type 4

**DOI:** 10.7759/cureus.105356

**Published:** 2026-03-17

**Authors:** Shamas Rafique, Aya Odeh

**Affiliations:** 1 Family Medicine, Family Medical Health Care PLLC, New York, USA; 2 Internal Medicine, First Affiliated Hospital of Xinjiang Medical University, Urumqi, CHN; 3 Cardiology, Family Medical Health Care PLLC, New York, USA; 4 Medicine and Surgery, Beewell International Hospital, Islamabad, PAK; 5 Medical Laboratory Science, College of Staten Island, New York, USA

**Keywords:** adult-onset sma, anterior horn cell disease, creatine kinase, electromyography, smn1, spinal muscular atrophy type 4

## Abstract

Spinal muscular atrophy (SMA) type 4 is a rare, adult-onset motor neuron disorder characterized by slowly progressive proximal weakness with preserved ambulation. Its indolent clinical course and nonspecific early manifestations frequently result in prolonged diagnostic delays. We present the complete diagnostic evaluation of a 33-year-old male patient with a multi-year history of painless, progressive lower extremity weakness. Initial laboratory testing revealed an isolated elevation of creatine kinase (CK) and vitamin D deficiency, with otherwise normal metabolic, endocrine, and inflammatory studies. Persistent CK elevation despite vitamin repletion prompted a neuromuscular referral.

Electrodiagnostic testing demonstrated normal nerve conduction studies with electromyographic evidence of widespread chronic active denervation and, critically, normal paraspinal musculature. This pattern strongly supported the localization of the pathology to the anterior horn cells. Subsequent genetic testing confirmed a homozygous deletion of exon 7 in the *SMN1* gene, establishing the definitive diagnosis of SMA type 4. This case underscores the importance of a systematic diagnostic approach integrating serial CK monitoring, electrodiagnostic localization, and genetic confirmation in evaluating adult-onset proximal weakness, particularly in the current era of disease-modifying therapies.

## Introduction

Spinal muscular atrophy (SMA) is an autosomal recessive neuromuscular disorder caused by a deficiency of the survival motor neuron (SMN) protein, leading to the degeneration of alpha motor neurons in the anterior horn of the spinal cord. The SMN protein is essential for the health and function of motor neurons. In more than 95% of cases, SMA results from a homozygous deletion of exon 7 in the SMN1 gene on chromosome 5q13 (the specific location of the gene on the long arm of chromosome 5). Disease severity and age of onset are modulated by the copy number of the nearly identical SMN2 gene, which produces limited amounts of functional SMN protein due to alternative splicing, a process that results in the production of different protein variants from a single gene [1-3].

SMA is clinically classified into types 1 through 4 based on age at onset and achieved motor milestones. SMA type 4 represents the adult-onset form, typically manifesting after age 18, with patients typically retaining the ability to walk and experiencing a slow progression of symptoms [4,5]. Adult-onset SMA type 4 is exceedingly rare, accounting for less than 5% of all SMA cases, and diagnostic delays of several years are common due to its indolent course and nonspecific presentation [6]. Because symptoms evolve insidiously over years, adult-onset SMA is frequently misdiagnosed as limb-girdle muscular dystrophy, inflammatory myopathy, radiculopathy, or even a functional neurological disorder [6].

This case report illustrates a complete diagnostic pathway culminating in genetic confirmation of SMA type 4. It emphasizes key clinical, laboratory, and electrodiagnostic features relevant to general internists and neurologists, highlighting the specific learning point that a systematic approach integrating serial creatine kinase (CK) monitoring, electrodiagnostic localization, and genetic confirmation can shorten this diagnostic odyssey.

## Case presentation

Clinical history

A 33-year-old previously healthy and physically active male patient presented with a five- to seven-year history of gradually progressive, painless bilateral lower extremity weakness. There was no preceding trauma, infection, or systemic illness. He reported increasing difficulty rising from low chairs, often requiring the use of his arms on his thighs (a modified Gower maneuver), and a growing reliance on handrails while climbing stairs. He reported progressive, symmetric proximal lower extremity weakness without distal involvement, consistent with a descending pattern of motor involvement. He denied any bulbar symptoms such as dysphagia or dysarthria, and there were no cardiorespiratory complaints like dyspnea or orthopnea. Over time, he developed intermittent paresthesias and insomnia, which he attributed to anxiety regarding his progressive, undiagnosed symptoms.

Physical examination

Serial examinations revealed a consistent pattern of symmetric proximal lower extremity weakness. Muscle strength, assessed by the Medical Research Council (MRC) scale, was graded at 4/5 in the bilateral hip flexors and knee extensors, while distal strength in the ankles and toes was preserved. Deep tendon reflexes were diminished at both the patellar and Achilles tendons. Sensory examination, coordination, and cranial nerve testing were all normal. Notably, the patient exhibited calf pseudohypertrophy. There was no evidence of muscle atrophy, fasciculations, spasticity, clonus, or other upper motor neuron signs. This pattern of symmetric proximal weakness with preserved distal strength and normal sensation is characteristic of motor neuron disorders and helped narrow the differential diagnosis.

Diagnostic workup and reasoning

The diagnostic evaluation spanned approximately eight months from initial presentation to genetic confirmation, with key intervals including initial laboratory testing (Month 0), vitamin D repletion trial (Months 0-1), neuromuscular referral (Month 1), MRI and electromyography (EMG)/nerve conduction studies (NCS) (Month 2), and genetic testing (Month 3).

The diagnostic journey began with a broad laboratory evaluation to screen for common causes of proximal myopathy. The differential diagnosis at presentation included limb-girdle muscular dystrophy, inflammatory myopathy (polymyositis), inclusion body myositis, radiculopathy, chronic inflammatory demyelinating polyneuropathy (CIDP), and motor neuron disease. Initial testing revealed a moderately elevated CK at 346 U/L (reference range 44-196 U/L) and a severe deficiency of 25-hydroxyvitamin D at 17 ng/mL (reference range 30-100 ng/mL). A comprehensive metabolic panel, thyroid-stimulating hormone, hemoglobin A1c, C-reactive protein, rheumatoid factor, and vitamin B12 levels were all within normal limits.

Given the vitamin D deficiency, high-dose repletion was initiated. However, at a follow-up appointment one month later, the patient reported no subjective improvement in his weakness. Repeat laboratory testing showed that while his vitamin D level had improved to 25 ng/mL, his CK remained persistently elevated at approximately 330 U/L. Notably, an aldolase level drawn at this time was normal at 5.2 U/L. This dissociation between a persistently elevated CK and a normal aldolase was a key clue, suggesting a neurogenic rather than a primary myopathic process. This finding, coupled with the failure of weakness to improve with vitamin D repletion, prompted a referral to a neurologist for further evaluation.

**Figure 1:**
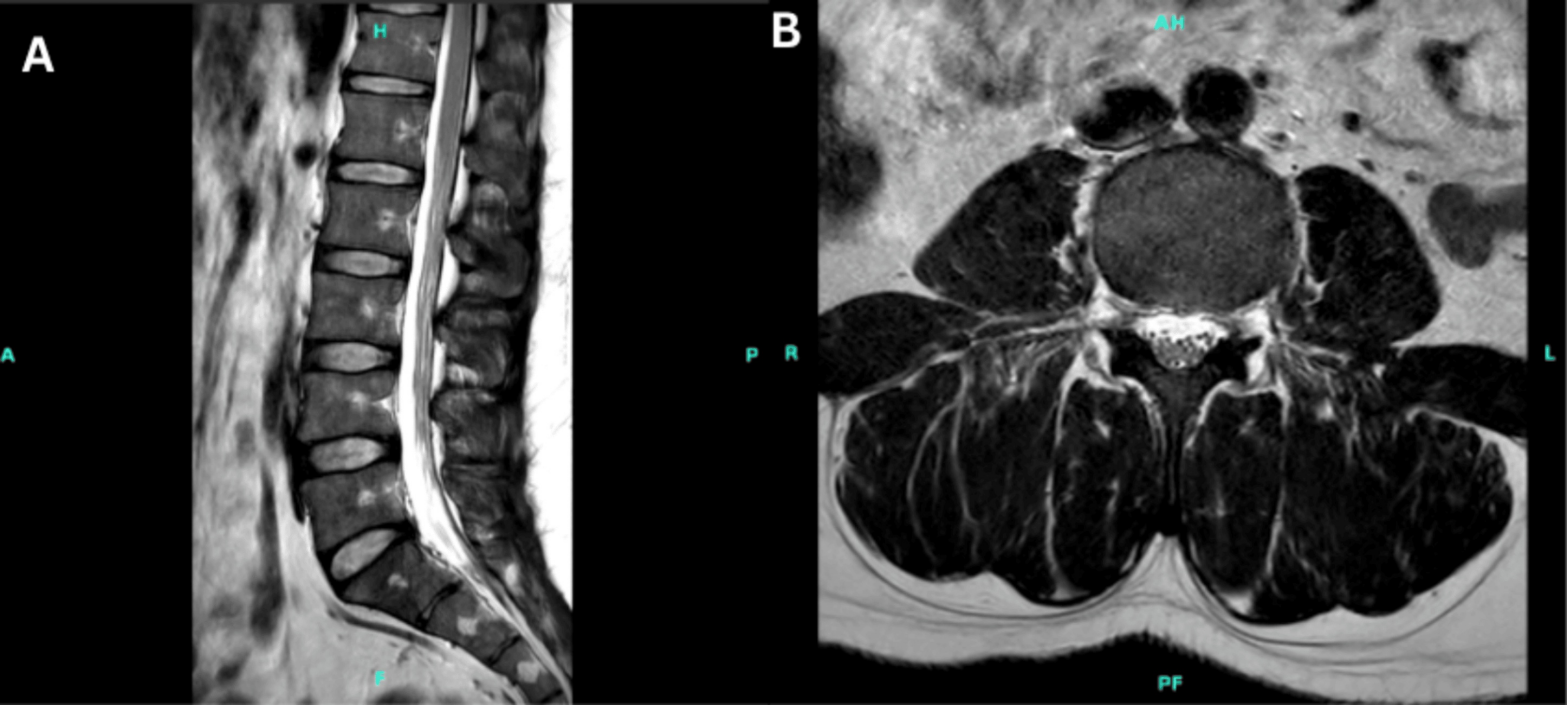
MRI of the Lumbar Spine WO (A) The axial T2-weighted image demonstrates a normal spinal canal, no disc herniation, and patent neural foramina. (B) Sagittal T2-weighted image shows normal alignment, preserved disc height, and no evidence of central canal stenosis or nerve root compression. These findings effectively exclude structural causes of radiculopathy.

MRI of the lumbar spine without contrast was performed to exclude structural causes of radiculopathy. Figure 1 (A: axial T2-weighted view and B: sagittal T2-weighted view) demonstrates normal findings with no evidence of disc herniation, central canal stenosis, foraminal narrowing, or nerve root compression. This further supported localization to the anterior horn cells rather than a radiculopathic process.

Electrodiagnostic studies (EMG/NCS)

Comprehensive NCS and needle EMG were performed to localize the site of the lesion. Sensory and motor NCS of multiple nerves in the upper and lower extremities revealed normal amplitudes, conduction velocities, and distal latencies, effectively ruling out a peripheral neuropathy or a disorder of the neuromuscular junction. Needle EMG examination revealed evidence of chronic active denervation in multiple muscles. In the lower extremities, the vastus lateralis, tibialis anterior, and medial gastrocnemius showed fibrillation potentials, positive sharp waves, and large-amplitude, long-duration motor unit potentials (MUPs) with reduced recruitment, indicating chronic denervation with reinnervation. Similar neurogenic changes were observed in upper extremity muscles, including the triceps and first dorsal interosseous, suggesting subclinical generalized involvement. A critical finding was the normal electrophysiology of the lumbosacral and cervical paraspinal muscles, which helped differentiate anterior horn cell disease from polyradiculopathy. These findings are summarized in Table 1.

**Table 1:** Summary of Needle Electromyography Findings MUPs: motor unit potentials

Muscle	Nerve/myotome	Key findings	Interpretation
Lower extremities			
Vastus lateralis	Femoral nerve (L2-L4)	Fibrillation potentials (+1), positive sharp waves (+1), large amplitude (6-8 mV), long-duration (15-18 ms) MUPs, reduced recruitment	Chronic active denervation
Tibialis anterior	Peroneal nerve (L4-L5)	Fibrillation potentials, positive sharp waves, large/long MUPs	Neurogenic pattern
Medial gastrocnemius	Tibial nerve (S1-S2)	Large amplitude MUPs, no spontaneous activity	Chronic neurogenic changes
Lumbosacral paraspinals	Multiple levels	Normal	Differentiates from radiculopathy
Upper extremities			
Triceps	Radial nerve (C6-C8)	Large/long MUPs, no spontaneous activity	Subclinical generalized involvement
First dorsal interosseous	Ulnar nerve (C8-T1)	Large/long MUPs, reduced recruitment	Neurogenic pattern
Cervical paraspinals	C5-C7	Normal	Supports anterior horn cell localization

With the pathology confidently localized to the anterior horn cells based on the clinical presentation and electrodiagnostic pattern, targeted genetic testing was pursued. Genetic confirmation via polymerase chain reaction (PCR)-based testing for homozygous deletion of exon 7 in the SMN1 gene remains the gold standard diagnostic method for 5q-associated SMA. This confirmed the clinical suspicion, revealing a homozygous deletion of exon 7 in the SMN1 gene. SMN2 copy number analysis demonstrated three copies, a genotype strongly associated with the milder, later-onset SMA type 4 phenotype (Table 2).

**Table 2:** Genetic Confirmation of Spinal Muscular Atrophy (SMA)

Gene tested	Result	Interpretation
*SMN1* exon 7	Homozygous deletion	Diagnosis of 5q-associated SMA
*SMN2* copy number	Three copies	Consistent with the SMA type 4 phenotype

Treatment and follow-up

Following the genetic confirmation, the patient received comprehensive counseling on the diagnosis, prognosis, and landscape of available disease-modifying therapies. After a multidisciplinary evaluation, he initiated treatment with risdiplam, an oral SMN2 splicing modifier. He was concurrently referred to physical therapy for a tailored strengthening and maintenance program. At his six-month follow-up, he reported a subjective stabilization of his previously progressive weakness. He continues to be followed longitudinally with serial functional assessments and CK monitoring to objectively evaluate his treatment response and disease trajectory. Longer-term follow-up with serial quantitative strength assessments, functional scores, and CK trends is ongoing and will be reported in future publications.

## Discussion

This case illustrates a classic yet frequently overlooked presentation of SMA type 4. The patient's diagnostic odyssey, spanning several years, highlights common clinical pitfalls. The diagnostic delay of 5-7 years in our patient mirrors that reported in other adult-onset SMA case series, where delays of 3-10 years are common due to misdiagnosis as muscular dystrophy or radiculopathy [6]. The insidious onset of proximal weakness in a young adult often prompts an initial search for inflammatory, endocrinologic, or muscular dystrophies. The persistent, moderate CK elevation observed here is a crucial and often misunderstood clue. While marked CK elevations are typical of muscular dystrophies, mild-to-moderate elevations can reflect the chronic denervation-reinnervation cycles occurring in motor neuron diseases and should always prompt a neuromuscular evaluation [7]. Furthermore, the dissociation between an elevated CK and a normal aldolase can serve as an early, subtle clue pointing away from a primary myopathy.

Electrodiagnostic studies provided the critical pivot point. Normal NCS effectively ruled out neuropathy and junctional disorders, while EMG revealed chronic neurogenic changes. The topographical diagnosis was refined by widespread changes in limb muscles contrasted with normal paraspinal musculature, a classic pattern strongly supporting anterior horn cell pathology and differentiating SMA from radiculopathies, which typically involve paraspinal muscles [8].

An atypical finding in our patient was calf pseudohypertrophy, which is uncommon in SMA type 4 and is more frequently associated with dystrophinopathies or limb-girdle muscular dystrophies. Pseudohypertrophy in neurogenic disorders is thought to result from relative sparing and compensatory hypertrophy of certain muscle groups, often type I fibers, or from pseudohypertrophy due to fatty infiltration and connective tissue replacement. Its presence in this case broadened the initial differential diagnosis but did not exclude SMA, as rare reports of pseudohypertrophy in adult-onset SMA exist. This finding underscores that atypical features do not rule out anterior horn cell disorders and should not delay genetic testing when the overall clinical and electrodiagnostic picture is supportive.

The confirmation of a homozygous SMN1 deletion with three SMN2 copies represents the phenotypic and molecular signature of SMA type 4 [9]. Our patient's presentation with proximal weakness, preserved ambulation, and three SMN2 copies is consistent with previously reported cohorts of SMA type 4 patients, in whom SMN2 copy numbers of 3-4 are typical and correlate with later onset and milder phenotype [9,10]. This diagnosis carries profound clinical implications that extend far beyond nomenclature. The treatment landscape for SMA has been revolutionized by the introduction of three disease-modifying therapies (nusinersen, risdiplam, and onasemnogene abeparvovec). Emerging data, including studies focused on adult populations, demonstrate that these therapies can stabilize or even improve motor function in later-onset forms of the disease [11-14]. This therapeutic reality fundamentally transforms the diagnostic imperative. For the patient in this case, the journey from unexplained symptoms to a precise molecular diagnosis was not merely an academic exercise; it was the critical first step toward accessing a therapy with the potential to alter his long-term prognosis and preserve his quality of life.

Limitations

As with any single case report, these findings should be interpreted within the context of the broader literature, and the diagnostic approach described should be validated in larger prospective studies. Clinical photographs and electrodiagnostic tracings were not available for publication, which limits the visual documentation of the calf pseudohypertrophy and the characteristic neurogenic changes described.

## Conclusions

SMA type 4 should be considered in the differential diagnosis for any adult presenting with progressive, painless proximal weakness, particularly when accompanied by a persistent mild-to-moderate CK elevation. A systematic, stepwise diagnostic approach is essential. This begins with initial laboratory testing to rule out common mimics, followed by targeted electrodiagnostic studies to identify patterns supportive of anterior horn cell pathology. The combination of diffuse chronic denervation on EMG with normal paraspinal muscles and normal NCS, in the appropriate clinical context, provides strong support for anterior horn cell localization. This then paves the way for definitive confirmatory genetic testing for SMN1 deletion. This case reinforces that a definitive diagnosis is not only achievable but also increasingly urgent in the modern therapeutic era, as it unlocks access to treatments that can fundamentally change the disease trajectory for patients with this rare but manageable condition.
